# The Pectin Methylesterase Gene Complement of *Phytophthora sojae*: Structural and Functional Analyses, and the Evolutionary Relationships with Its Oomycete Homologs

**DOI:** 10.1371/journal.pone.0142096

**Published:** 2015-11-06

**Authors:** Brent B. Horowitz, Manuel D. Ospina-Giraldo

**Affiliations:** Biology Department, Lafayette College, Easton, Pennsylvania, United States of America; National University of Ireland - Galway, IRELAND

## Abstract

*Phytophthora sojae* is an oomycete pathogen that causes the disease known as root and stem rot in soybean plants, frequently leading to massive economic damage. Additionally, *P*. *sojae* is increasingly being utilized as a model for phytopathogenic oomycete research. Despite the economic and scientific importance of *P*. *sojae*, the mechanism by which it penetrates the host roots is not yet fully understood. It has been found that oomycetes are not capable of penetrating the cell wall solely through mechanical force, suggesting that alternative factors facilitate breakdown of the host cell wall. Pectin methylesterases have been suggested to be important for *Phytophthora* pathogenicity, but no data exist on their role in the *P*. *sojae* infection process. We have scanned the newly revised version of the annotated *P*. *sojae* genome for the presence of putative pectin methylesterases genes and conducted a sequence analysis of all gene models found. We also searched for potential regulatory motifs in the promoter region of the proposed *P*. *sojae* models, and investigated the gene expression levels throughout the early course of infection on soybean plants. We found that *P*. *sojae* contains a large repertoire of pectin methylesterase-coding genes and that most of these genes display similar motifs in the promoter region, indicating the possibility of a shared regulatory mechanism. Phylogenetic analyses confirmed the evolutionary relatedness of the pectin methylesterase-coding genes within and across *Phytophthora* spp. In addition, the gene duplication events that led to the emergence of this gene family appear to have occurred prior to many speciation events in the genus *Phytophthora*. Our results also indicate that the highest levels of expression occurred in the first 24 hours post inoculation, with expression falling after this time. Our study provides evidence that pectin methylesterases may be important for the early action of the *P*. *sojae* infection process.

## Introduction

The common soybean, *Glycine max*, is a widespread food crop both in the United States and around the world. In the United States alone approximately 84 million acres of soybean crop were planted in 2014, with an estimated production value of $40 billion (http://www.nass.usda.gov/Statistics_by_Subject/result.php?FE19E06A-4BD2-3372-8CE4-2A50B0699BF6&sector=CROPS&group=FIELDCROPS&comm=SOYBEANS). Numerous maladies affect *G*. *max*, with at least 33 different diseases reported in 1996 in North America alone [1].

*Phytophthora sojae* is an oomycete plant pathogen causing root rot in *G*. *max*. Worldwide, *P*. *sojae* is responsible for almost $2 billon dollars in annual losses, with $200 million reported in the northern Midwest of the U.S. alone [2]. Preventing *P*. *sojae* infection is made difficult by the rapid rate of mutation exhibited by the pathogen, which allows quick adaptation to new forms of infection control; additionally, as an oomycete, *P*. *sojae* is not affected by most fungal treatments [2].

As a hemibiotrophic organism, *P*. *sojae* starts its infection process with biotrophic activity but transitions into a necrotrophic stage shortly after infection and uses the decaying plant tissue as an energy source. Initial infection is achieved in part by the adhesion of a spore to the root surface and formation of a germ tube [3]. The germ tube has an appendage termed an appressorium, which uses turgor pressure to attempt to breach the host cell wall.

Several oomycete species, including *P*. *sojae*, have had their genomes sequenced and annotated. This wealth of genomic information, which has recently become available, has led to great advances in understanding the disease cycle of *P*. *sojae*. Much work has centered on genes encoding effector proteins, which tend to modulate the plant defense response. Such proteins are characterized by the presence of an amino acid motif with the sequence RxLR, which was first discovered in the genomes of *P*. *sojae* and *P*. *ramorum* and has since been suggested to be essential to the entry of the pathogen into the cell [4], although this remains controversial [5–8].

Because of its potential role in oomycete pathogenicity, a group of genes encoding enzymes involved in carbohydrate metabolism has also been the target of multiple of investigations [9]. These carbohydrate-active enzymes (also known as CAZymes) are classified into superfamilies (each composed of multiple families) referred to as Glycoside Hydrolases (GH), Glycosyl Transferases (GT), Polysaccharide Lyases (PL), or Carbohydrate Esterases (CE) [10]. Of these groups of enzymes, the GH, PL, and CE superfamilies appear to be implicated in cell wall degradation [9]. As the appressorium cannot pierce the cell wall using its turgor pressure alone [11], it has been suggested that these cell wall degrading enzymes (CWDE) may also play an important role in pathogenesis [9].

Among the most prominent components of the cell wall is the polysaccharide pectin, which can account for 35% of cell wall dry mass [12]. Pectin is a large molecule consisting of as many as seventeen distinct monosaccharides, with a large contingent of galacturonic acid [13]. Within pectin, linear polymers of α-(1,4)-galacturonic acid residues are often modified at the C-6 carboxyl end with a methyl group added via an ester bond. The breakdown of this methylester modification is catalyzed by pectin methylesterases (PME), a group of enzymes classified as family 8 of the CE superfamily. Breakdown results in the release of methanol, protons, and the remainder of the polysaccharide, which is then open to other enzymatic actions [13]. Pectate lyase and polygalacturonase, members of the PL and GH superfamilies respectively, are among other enzymes contributing to the breakdown of pectin. These pectin-degrading enzymes are among the most represented CWDE in the *Phytophthora* genomes [9]. PME activity by plant pathogens has also previously been indicated to be important for successful infection [14].

A previous study in *Phytophthora infestans*, a pathogen of potato and tomato, revealed that PME expression in that species is highest in the first day of infection [15]. In contrast, analysis of PME expression in *Phytophthora capsici* revealed that highest levels of expression occur after one week of infection [16]. Life cycle differences among *P*. *infestans*, *P*. *sojae*, and *P*. *capsici* (as well as the biology of their hosts) might give rise to the possibility that the three *Phytophthora* species express pectin methylesterase genes at different time points.

In order to understand the role pectin breakdown plays during the infection cycle of *P*. *sojae*, we scanned the newly revised version of the annotated *P*. *sojae* genome for the presence of putative pectin methylesterases genes and conducted a comprehensive sequence analysis of all gene models found. In addition, we analyzed the PME gene expression levels in *P*. *sojae* throughout the early course of infection of susceptible soybean plants.

## Methods

### Cultures

#### Glycine max

Seeds from *Glycine max* cv. Williams, which is susceptible to *P*. *sojae* infection, were grown in Miracle Gro® Potting Soil at 24°C with a 16h light:8h dark cycle and watered daily. Plants were grown for three weeks.

#### Phytophthora sojae

Cultures of *P*. *sojae* race R5 were acquired from the United States Department of Agriculture in Beltsville, Maryland and stored on Rye A agar [17]. Isolates were also grown on unclarified V8®/Lima Bean agar for infection assays [18]. Nucleic acid extraction was done from *in vitro* mycelial cultures grown on pea broth (120g of frozen peas autoclaved, supplemented with 2g CaCO_3_ and 0.05g β-sitosterol in 1L water) at 22°C in the dark.

### Infection assay and RNA extraction

A 2mm^2^ plug from an unclarified V8®/Lima Bean agar culture was inserted into a lesion in the soybean hypocotyl. Leaf tissue samples were collected at 12, 24, 36, 48, and 72 hours post inoculation (hpi). Samples were flash frozen in liquid nitrogen and stored at -80°C. RNA was extracted from the leaf tissue using the Qiagen RNeasy Mini RNA kit (Qiagen, Valencia, CA). The extraction process included an on-column DNase cleanup step. RNA integrity was confirmed via gel electrophoresis.

#### PME gene selection in *P*. *sojae*

The CAZyme database (http://www.cazy.org/) was searched for annotated sequences of PME genes belonging to fungi or *Arabidopsis thaliana* (at the time this research was conducted no oomycete PME gene sequences were available in the CAZyme database). Obtained sequences were used to perform BLAST searches for *P*. *sojae* PME-encoding genes in the DOE Joint Genome Institute (http://genome.jgi-psf.org/Physo3/Physo3.home.html) and FungiDB (http://fungidb.org/fungidb/?nobounce) genome databases. For expression analysis, only sequences meeting the following criteria were examined: E-values smaller than 1x10^-10^, sequence length between 500 and 2000bp, fewer than four introns, no introns exceeding 250bp, and canonical start and stop codons.

### Primer Design

All PME genes were aligned in MEGA6 by the ClustalW method [19]. Primers were designed targeting regions with the greatest level of sequence difference among all genes (S1 Table). Mismatches to other areas of the *P*. *sojae* genome or to the *G*. *max* host were checked with the NCBI Primer BLAST system in order to prevent unintended amplification of other *P*. *sojae* or *G*. *max* sequences.

### Intron Analysis

PCR primers were additionally designed in order to determine the validity of an unusually large intron observed in gene model 339170. The specific reverse primer originally designed to amplify the full gene sequence was used in combination with one of two different forward primers, depending on the template: for amplification using cDNA as a template, a forward primer originating in the 3’ end of the exon immediately preceding the intron and continuing into the 5’ end of the second exon flanking the intron, was used. For amplification using gDNA, a different forward primer, which began in the 3’ end of the first exon and continued into the intron, was used.

### Reverse Transcription and Quantitative PCR

Qiagen’s Quanti-Tect Reverse Transcription Kit (Qiagen, Valencia, CA) was used to create cDNA libraries from both the *in planta* and *in vitro* RNA samples. This cDNA was then quantified on an iQ5 Real Time PCR Detection System (Bio-Rad, Hercules, CA). Results were analyzed for relative expression by Livak’s ΔΔC_t_ method using actin as the constitutive reference gene [20]. Data are the average of three technical replicates. Data visualization was achieved using the Multiexperiment Viewer application (http://www.tm4.org/mev.html), which is part of the microarray software suite TM4 [21].

### Phylogenetic Analysis

The oomycete genomes available on the FungiDB database [*P*. *capsici* (PHYCA), *P*. *cinnamomi* (PHYCI), *P*. *infestans* (PITG), *P*. *parasitica* (PPTG), *P*. *ramorum* (PSURA), *P*. *sojae* (PHYSO), and *Hyaloperonospora arabidopsidis* (Hara)] were BLAST-searched for putative PME genes, using the reference sequences retrieved from the CAZyme database. The putative genes thus obtained were curated by E-value from the BLAST search (smaller than 1x10^-10^), transcript length (500-2000bp), intron count (three or fewer), and canonical start and stop codons. DNA (protein-coding) sequences were aligned using ClustalW. Subsequently, the Maximum-Likelihood method was utilized to construct phylogenies [22], with 10,000 bootstrap replications for validity testing using MEGA6 [19].

### Bioinformatic analyses

A MEME analysis (http://meme-suite.org/) [23] was performed on the 500bp segment immediately upstream of the start codon for each oomycete sequence, in order to search for putative promoter motifs with potentially regulatory functions. Sequences not containing the entire 500bp segment were not used in this analysis.

In order to determine whether the deduced proteins could be secreted, the sequences for all *P*. *sojae* PME genes were analyzed with SignalP 4.1 (http://www.cbs.dtu.dk/services/SignalP/), using the sensitive cutoff values [24]. The D-cutoff values, which are used to discriminate signal peptides from non-signal peptides, are reported. Also reported are the cleavage site based on the raw C-score (C position), the position where the maximum signal peptide score occurs (S position), and the position for the maximum Y-score (Y position), which is obtained based on the C-score and the S-score, and results in a better cleavage site prediction than the raw C-score alone [24].

## Results

### Putative PME gene complement in *P*. *sojae*

Using reference genes from *Aspergillus nidulans*, *Fusarium fujikuroi*, and *Arabidopsis thaliana* obtained from the CAZyme database, 16 candidate genes were found in the *P*. *sojae* genome, which were chosen for further study based upon the criteria listed above (Table 1). All of the 16 studied genes contained a PFAM domain for pectin methylesterase according to the JGI annotation. BLAST analyses revealed seven other putative genes, of which six were discarded for lack of canonical start or stop codon; the seventh gene model was not used because its transcript length (480 bp) did not meet the minimum specified. This gene model also lacked the PFAM domain for pectin methylesterase.

**Table 1:** *Phytophthora sojae* PME-coding genes selected for this study.

Gene ID	Transcript length (bp)^§^	Scaffold	Location*	Introns^‡^
245865	1035	3	3844703–3845737 (-)	0
257384	735	1	1880430–1881164 (-)	0
257416	1020	1	1878823–1879842 (+)	0
257622	1050	1	1857315–1858364 (-)	0
260992	921 (713, 208)	8	1317570–1318600 (+)	1 (110bp)
339170	888 (8, 82, 512, 286)	8	1213079–1214328 (-)	3 (236bp, 50bp, 76bp)
339194	1017 (963, 81)	8	1283698–1284746 (+)	1 (32bp)
340202	852 (156, 365, 331)	9	1281435–1282379 (-)	2 (28bp, 65bp)
340204	846 (420, 324, 102)	9	1290916–1291893 (-)	2 (83bp, 49bp)
468280	1020 (522, 498)	1	1853308–1854407 (-)	1 (80bp)
491908	1032	3	3641997–3643028 (-)	0
520304	1029	8	1310707–1311735 (-)	0
520405	1044	8	1400437–1401480 (+)	0
522637	645	8	1328913–1329557 (-)	0
523081	1029	9	1283881–1284909 (-)	0
528421	1029 (531, 498)	11	934962–936053 (+)	1 (63bp)

(^§^) Exon lengths

(*) strand,and

(^‡^) intron sizes are given in parenthesis.

### Pectin methylesterase expression during infection

Expression for all tested genes was detected both *in vitro* and i*n planta*, except for gene model 260992, which indicates that this gene is not expressed by *P*. *sojae* during infection. The primers for this transcript successfully amplified *P*. *sojae* genomic DNA, and expression was also found in mycelium.

**Fig 1:**
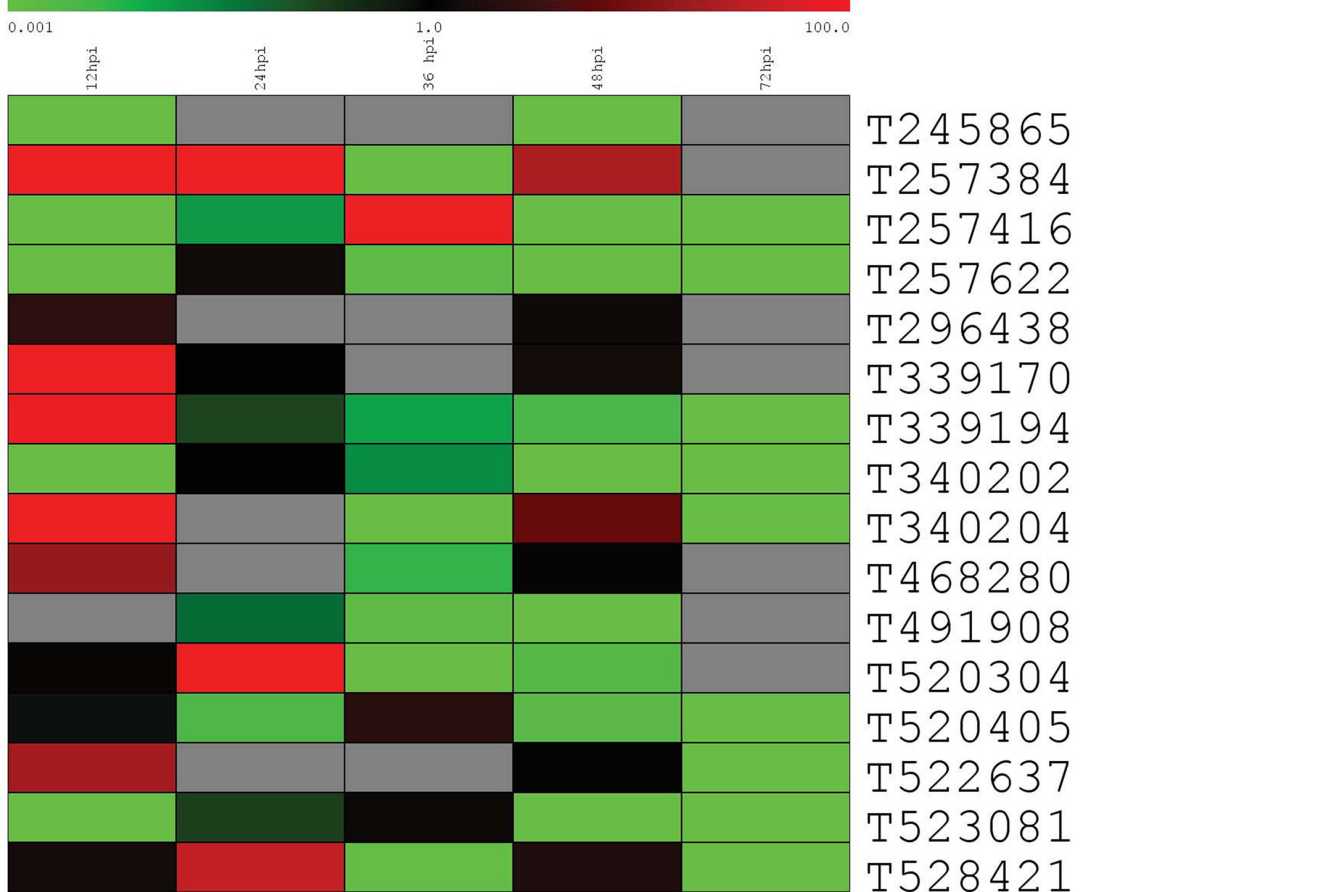
Relative expression data at five time points post-inoculation obtained by analysis of qPCR data using Livak’s method. Actin was used as a reference gene, and the calibrating sample was mycelial RNA. Red indicates up-regulation, green down-regulation, black neutral, and grey indicates transcription was not detected. Columns represent time-points, rows are transcript ID numbers.

Using Livak’s method [20], relative expression rates were calculated; differential expression trends are displayed as a heatmap (Fig 1). Most genes were up-regulated in the host soybean. Based on the actual expression rates, eight of the sixteen genes were at their highest levels of expression at 12hpi, while five genes were highest at 24hpi and three were highest at 36hpi (S2 Table). Four genes did not appear to change expression in a substantial way. By 72 hpi, all genes that were still detected were down-regulated at least 100-fold when compared with mycelial levels.

### Phylogenetic analysis

**Fig 2:**
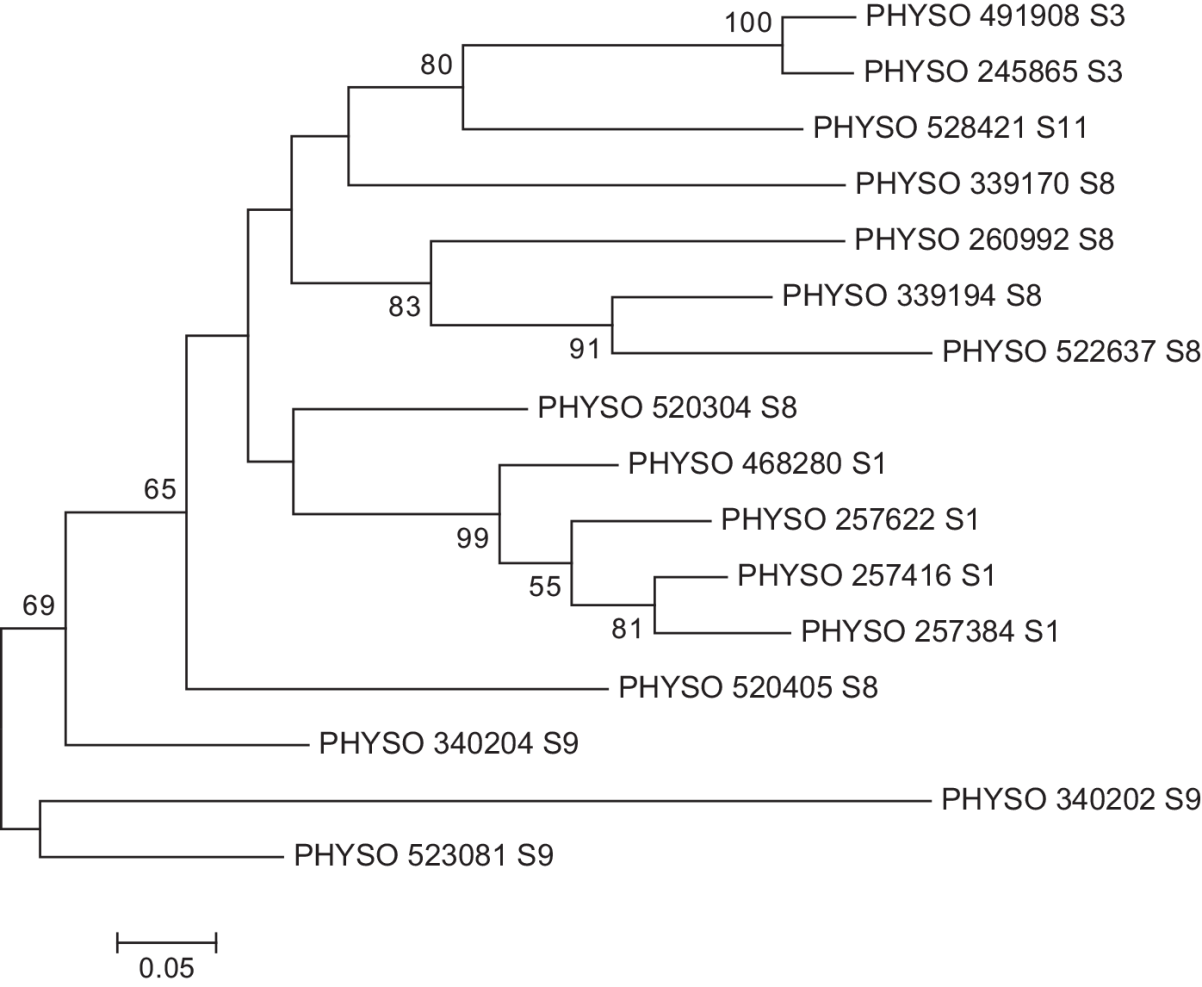
Phylogenetic analysis of the PME gene complement of *P*. *sojae*, including scaffold location for each gene model. Analysis was conducted using 16 DNA sequences as listed in the DOE Joint Genome Institute database for *P*. *sojae* (S1 Appendix). The evolutionary history was inferred using the Maximum-Likelihood method [22] and the tree with the highest log likelihood is shown. Robustness of the tree was evaluated by a bootstrap test with a 10,000 replications [25] and the percentage of trees in which the associated taxa clustered together is shown next to the branches (bootstrap values lower than 50% are not displayed). The tree is drawn to scale, with branch lengths measured in the number of substitutions per site. The analysis involved 16 nucleotide sequences. Partial deletion was used and all positions with less than 95% site coverage were eliminated. That is, fewer than 5% alignment gaps, missing data, and ambiguous bases were allowed at any position. There were a total of 293 positions in the final dataset. Scale bar: number of substitutions per site. Labels include the unique *P*. *sojae* gene identifier and the scaffold (S) where each gene model is located. Evolutionary analyses were conducted in MEGA6.

Sequences of the *P*. *sojae* gene models whose expression was tested by qPCR were aligned by ClustalW and a phylogenetic tree was constructed using the Maximum-Likelihood method (Fig 2). For each operational taxonomic unit its genomic location, or scaffold, is given. The phylogenetic tree consisted of two major clades, one of them containing the majority of the *P*. *sojae* PME gene models. Genes within the same scaffold were positioned together in the same clade (excepting scaffold 8 genes 339170 and 520304). Furthermore, the bootstrap analysis supports the hypothesis that at least six of these gene models can be grouped in three pairs of true paralogous genes.

The genomes of 19 oomycetes were searched in FungiDB by BLAST using the same sequences previously described for putative pectin methylesterases. The genomes of six species of *Phytophthora* (*P*. *capsici*, *P*. *cinnamomi*, *P*. *infestans*, *P*. *parasitica*, *P*. *ramorum*, and *P*. *sojae*) and *H*. *arabidopsidis* were found to contain putative pectin methylesterase genes. Conversely, six species of *Pythium* (*P*. *aphanidermatum*, *P*. *arrhenomanes*, *P*. *irregulare*, *P*. *iwayamai*, *P*. *ultimum*, and *P*. *vexans*), two species of *Saprolegnia* (*S*. *declina* and *S*. *parasitica)*, two species of *Albugo* (*A*. *candida* and *A*. *laibachii*) and two species of *Aphanomyces* (*A*. *astaci* and *A*. *invadans*) did not return any significant results in the BLAST analysis. An additional search of the annotated genomes using the EC number (3.1.1.11) similarly did not provide any putative sequences from those twelve species, but retrieved the same sequences from the *Phytophthora* spp. and *H*. *arabidopsidis*.

**Fig 3:**
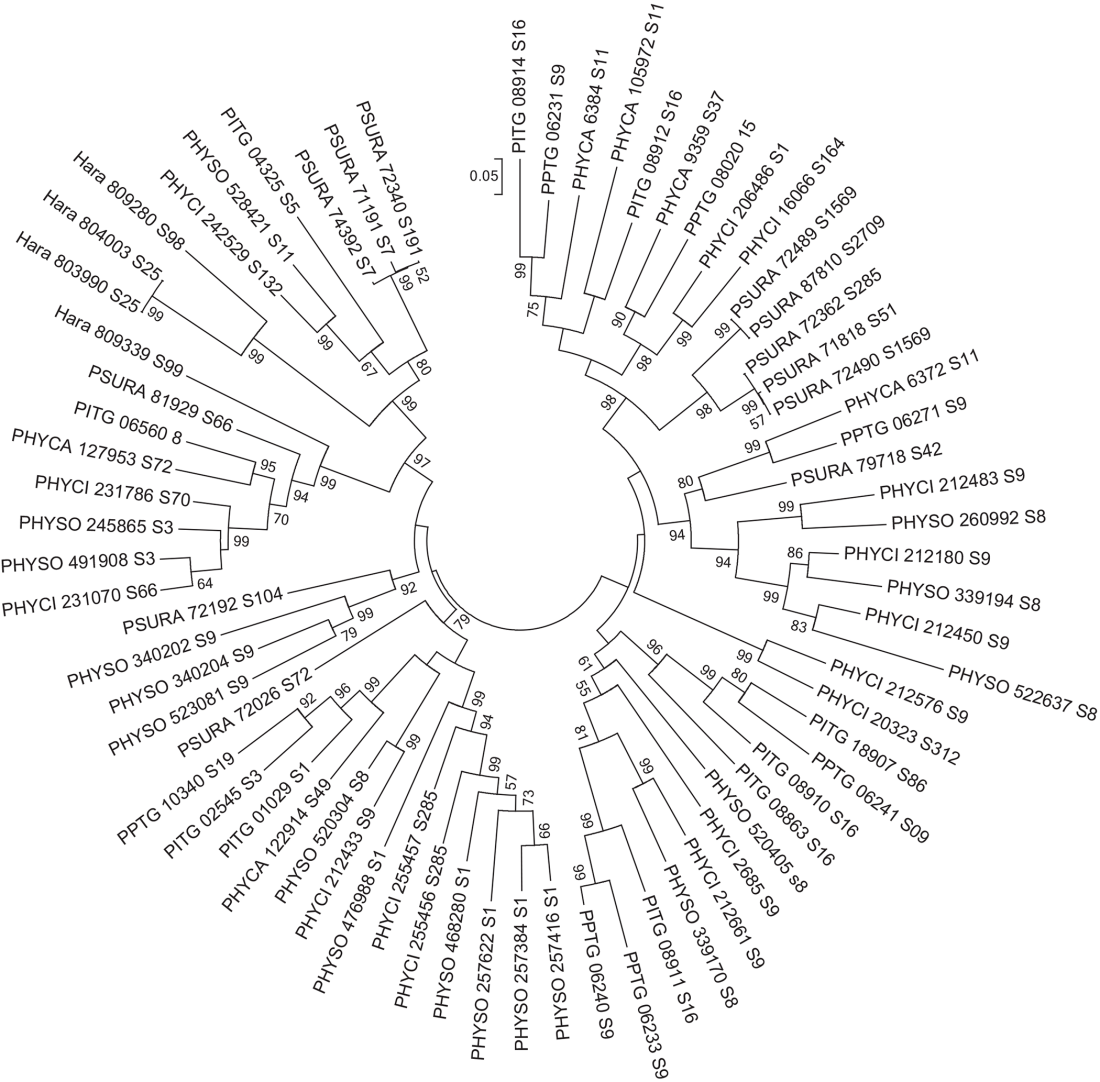
Phylogeny of the PME gene complements in the oomycetes. Analysis was conducted using 71 protein-coding DNA sequences obtained from different database sources (S2 Appendix). The evolutionary history was inferred using the Maximum-Likelihood method [22] and the tree with the highest log likelihood is shown. Robustness of the tree was evaluated by a bootstrap test with a 10,000 replications [25] and the percentage of trees in which the associated taxa clustered together is shown next to the branches. The tree is drawn to scale, with branch lengths measured in the number of substitutions per site. Bootstrap values lower than 50% are not displayed. Partial deletion was used and all positions with less than 95% site coverage were eliminated. That is, fewer than 5% alignment gaps, missing data, and ambiguous bases were allowed at any position. There were a total of 1203 positions in the final dataset. Scale bar: number of substitutions per site. Labels are as follows: *P*. *capsici* (PHYCA), *P*. *cinnamomi* (PHYCI), *Phytophthora infestans* (PITG), *P*. *parasitica* (PPTG), *P*. *ramorum* (PSURA), *P*. *sojae* (PHYSO), and *Hyaloperonospora arabidopsidis* (Hara), followed by transcript ID and scaffold number. Evolutionary analysis was conducted in MEGA6.

Phylogenetic analyses using all oomycete PME protein-coding DNA sequences available (*P*. *capsici*, *P*. *cinnamomi*, *P*. *infestans*, *P*. *parasitica*, *P*. *ramorum*, *P*. *sojae*, and *H*. *arabidopsidis*) confirmed the evolutionary relatedness of PME-coding genes in the oomycetes (Fig 3). Each of the four major clades identified after the phylogenetic analysis included sequences from all *Phytophthora* spp., but sequences from *H*. *arabidopsidis* were restricted to only one clade. Twelve of the 16 *P*. *sojae* sequences were clustered in two of the clades. Several instances of paralogous genes were observed in each clade, mostly in the *P*. *cinnamomi*, *P*. *ramorum*, *P*. *sojae*, and *H*. *arabidopsidis* genomes.

### Promoter and signal peptide analysis

**Fig 4:**
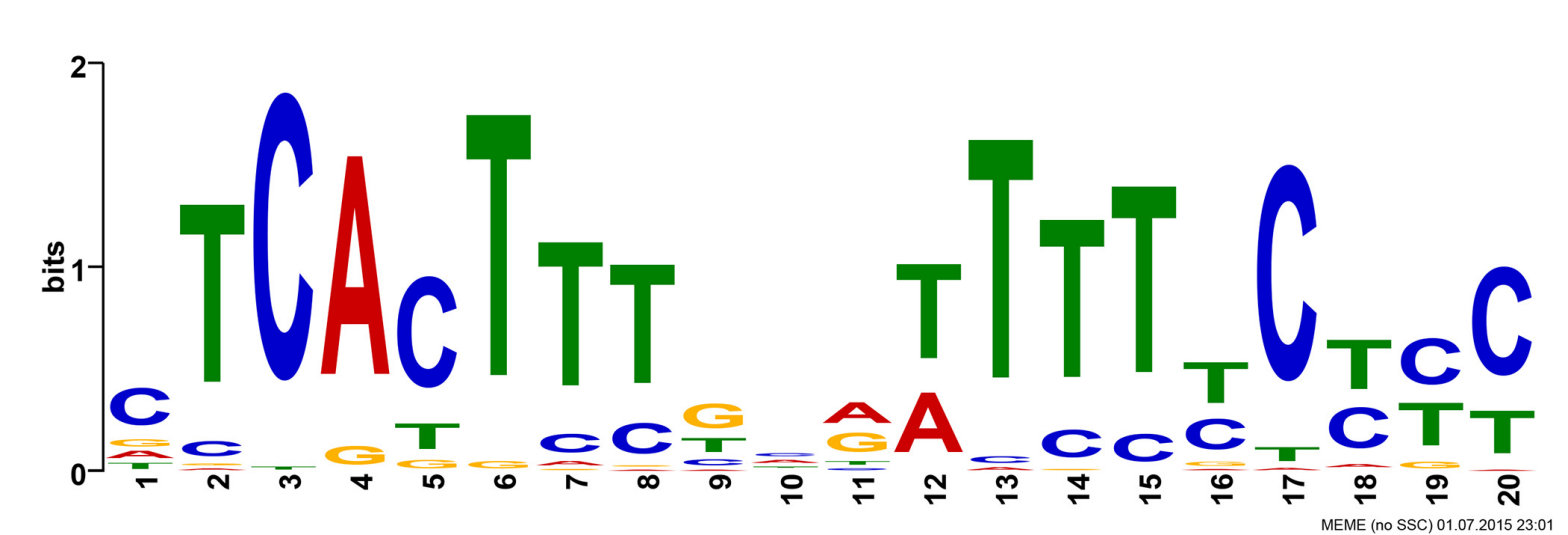
Shared motif in the PME promoter sequence region. The motif is located within a 500bp segment upstream of the translational start point of 59 of 68 putative oomycete PME genes. Size of base indicates relative level of conservation in the examined sequences.

The MEME suite was used to analyze the 500 bases immediately upstream of the start codon of the oomycete PME genes. In 59 of 68 PME sequences analyzed, a conserved motif was located (Fig 4). SignalP indicated that all PME transcripts from *P*. *sojae* considered for expression analysis also contained a putative peptide cleavage site signal (Table 2).

**Table 2:** Summary of SignalP 4.1 findings. C, S, Y, and D are values as defined in the methods above.

Gene ID	C position	S position	Y position	D-cutoff	False Positive Rate
245865	25	8	25	0.604	0.003
257384	41	14	26	0.580	0.003
257416	23	14	23	0.594	0.003
257622	26	8	26	0.501	0.003
260992	26	8	26	0.528	0.003
339170	17	13	17	0.682	0.003
339194	25	7	25	0.644	0.003
340202	22	42	22	0.437	0.009
340204	29	7	29	0.598	0.003
468280	17	14	17	0.577	0.003
491908	25	8	25	0.546	0.003
520304	35	8	23	0.528	0.003
520405	22	7	22	0.478	0.009
522637	21	2	21	0.374	0.009
523081	30	13	19	0.419	0.009
528421	38	10	22	0.348	0.009

### Intron analysis

**Fig 5:**
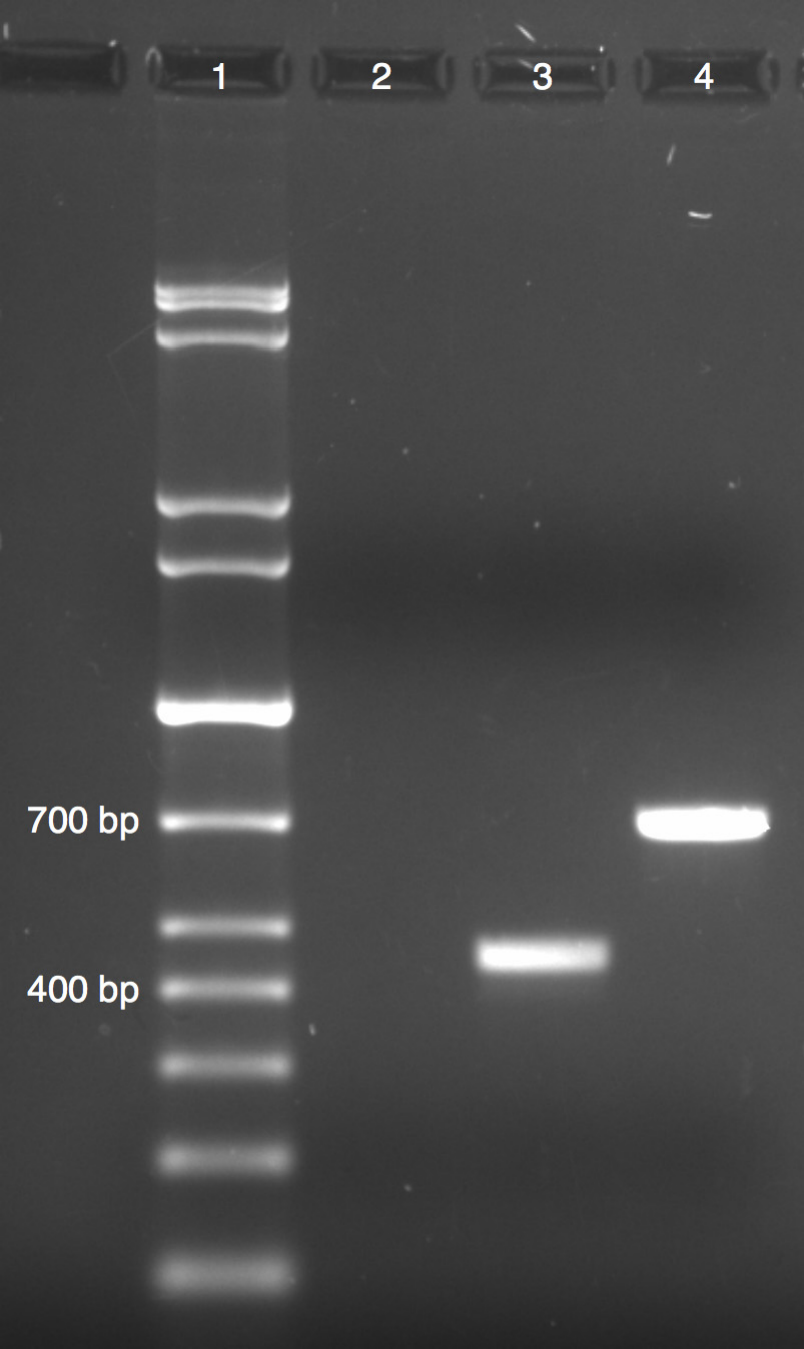
PCR results from intron analysis for gene 339170. Amplification with cDNA as template was conducted with primers 339170F12E and 339170R; amplification with genomic DNA was performed with primers 339170F1IE and 339170R. Expected amplicon lengths were 401bp and 687bp, respectively. Labels are as follows: Lane 1: 1 kb DNA marker; Lane 2: negative (minus reverse transcriptase) control; Lane 3: cDNA product; Lane 4: gDNA product.

Because of the relative paucity in the literature regarding experimental evidence on the presence and nature of introns in *Phytophthora* spp., we decided to further investigate the validity of a comparatively large intron predicted in the PME gene model 339170. PCR amplification, using cDNA and gDNA as templates, confirmed the presence of the first intron in this gene (Fig 5). This intron, beginning at base 9 of the coding region, extends to base 245 and is particularly large for an intron within *Phytophthora* [26].

## Discussion

Previous analyses based on the first annotated version of the *P*. *sojae* genome had indicated the potential existence of up to 19 PME homologs in this oomycete [15]. However, since that report, a revised, more complete annotated version of the *P*. *sojae* genome has become available and new strategies and tools, such as the FungiDB database, have been developed to search and find homologs and sequence motifs. According to our results, the number of *P*. *sojae* PME gene models that meet minimum criteria for functionality, in particular the presence of a PFAM domain for pectin methylesterase, has been reduced to 16, which still is a very large number for a gene family, and equals the size of CE family 5 (cutinases) reported elsewhere [27]. These 16 gene models are distributed in five scaffolds, with scaffolds 1 and 8 (each with five homologs) containing the majority of PME gene models found in *P*. *sojae*.

qPCR results indicate that all tested genes were expressed *in planta* (Fig 1), with the exception of gene model 260992, whose transcriptional activity was detected in mycelium only. The promoter for this gene appears to be intact and contains the 20bp motif found in other 58 (out of 68) PME promoter sequences (see below). All genes reached their highest level of up-regulation within 36hpi, indicating the synthesis of PME products was of the greatest importance at the start of the infection process. In fact, by 12hpi, at least six genes had been expressed at maximum levels. The importance of profiling gene expression at early infection stages, when the pathogen is in the active process of penetrating the host plant cell has been previously emphasized in *P*. *infestans* studies [28]. The results of our investigations focusing on the transcriptional changes occurring at that early stage in *P*. *sojae* are consistent with the evidence found in previous studies using *in vitro* and *in planta* approaches with *P*. *infestans* [15]. Furthermore, they confirm the substantial switch in gene expression occurring once the pathogen transitions to the necrotrophic stage. As pectin has been shown to be an early target for degradation by other plant pathogens, it is reassuring to find such a pattern in *P*. *sojae* [29]. Further, all genes that were still detectable at 72hpi were down-regulated relative to their *in vitro* expression levels. These data, when taken together, indicate that pectin breakdown is central to the life cycle of *P*. *sojae* while it is in its earlier biotrophic phase, but of lesser importance in the necrotrophic phase [2].

A phylogenetic tree of the *P*. *sojae* PME gene models shows that the sequences located on the same scaffold cluster together (Fig 2). This is seen most strongly with the genes on scaffold 1 and 9, but also perceived on scaffolds 3 and 8. Taken together, this suggests multiple events of gene duplication in the evolutionary history of *P*. *sojae*, where the PME genes could have been duplicated by slippage, a mechanism *Phytophthora* has previously been indicated to possess [30]. Indeed, genes found on the same scaffold are consistently located within relatively short distances from one another; for instance, the four genes on scaffold 1 are located in a 28kb section (Table 1). In addition, the proximity of the gene sequences and the presence of shared sequence motifs (see below) would indicate that a mechanism for coordinated regulation is likely to exist.

Neither of the genes on scaffold 3 (245865 and 491908) was found to be up-regulated at any tested point relative to the levels in the mycelium. Conversely, all genes on scaffold 1 were found to have elevated expression at one or more time points. While a separate analysis is needed to confirm this, one possible explanation for this finding is that chromatin structure around certain PME genes is heavily involved in controlling gene expression [31]. Chromatin structure could either hinder or allow expression in an area, limiting it on scaffold 3 but allowing scaffold 1 PME genes to be expressed. Examination of local chromatin structures would confirm or reject this hypothesis.

The phylogenetic analysis conducted with all available oomycete PME sequences revealed that there are multiple examples of paralogous genes in each clade. Surprisingly, no strong support for paralogy was observed in the cases of *P*. *capsici*, *P*. *infestans*, and *P*. *parasitica* genomes, despite the large PME gene complements these species have. This suggests that perhaps most (if not all) of the PME genes found in these *Phytophthora* spp. may predate their speciation events. We have previously reported that in *Phytophthora* spp. general chromosomal organization seems to be conserved [9, 32] and that synteny (or more appropriately, collinearity), understood as the similarity in chromosomal location of two homologous genes (likely to be orthologs) from different species, can be frequently observed. This has been the case for the genomes of *P*. *sojae* and *P*. *ramorum* and has been confirmed in our studies, in which the synteny between *P*. *sojae* and *P*. *cinnamomi* has also been revealed. Furthermore, our comprehensive phylogenetic analysis of all available PME sequences within the phylum Oomycota provided additional evidence toward several gene duplication events taking place throughout the evolutionary history of the phylum. Notably, each sequence from scaffold 8 in *P*. *sojae* clusters with a sequence from scaffold 9 in *P*. *cinnamomi* more closely than with any other sequence in *P*. *sojae*, which would facilitate the assignment of true orthology between *Phytophthora* spp. This provides additional evidence that those two scaffolds are homologous, and that, as mentioned above, the proposed gene duplication events in the PME genes occurred before the divergence of the two species. If this duplication hypothesis is correct, it would also suggest the existence of physical chromosomal proximity between unlinked scaffolds; for example, five putative PME genes located on several small scaffolds of the *P*. *ramorum* genome sequence, cluster together in the phylogenetic tree. Given that most oomycete species shown here have PME genes in close proximity, it would appear likely that those scaffolds are similarly closely attached and part of the same chromosome.

Of the twelve oomycete genomes without any putative PME genes, four are pathogens of animals (*A*. *astaci*, *A*. *invadans*, *S*. *declina*, and *S*. *parasitica*). *Pythium* species may not have PME activity inherently, but rather depend upon host PME activity, which could explain why no putative PME genes in those genomes were found [33]. It has also been previously indicated that *Pythium* does not have the same mechanisms for pectin breakdown that *Phytophthora* uses [34]. Searches of the transcriptional complements of both *Albugo spp*. obtained from FungiDB (*A*. *candida* and *A*. *laibachii*) using the CAZymes Analysis Toolkit (http://bobcat.ornl.gov/besc/index.jsp, [35]) similarly did not reveal the presence of any putative PME genes. Previous studies on *Albugo* have not focused on its CAZyme complement but it has been indicated that *Albugo* spp. and *H*. *arabidopsidis*, the obligate biotrophs analyzed here, have developed different mechanisms for metabolism, and that *Albugo* in particular avoids host defense by not hydrolyzing the cell wall [36]. Carbohydrate fragments are particularly strong activators of host immunity, and an overall decrease in cell wall degradation has been observed in obligate plant pathogenic oomycetes [37].

Searching the 500 bp upstream of the start codon revealed a 20bp motif in 59 of 68 tested PME sequences (Fig 4). This motif is most often found within the 50 bases before the start codon, although in 8 of the analyzed sequences the motif occurred further upstream. The motif, CTCACTTTKNRWTTTTYCYYY, bears resemblance to the previously located motif GCTCATTYYNCAWTTTT hypothesized to be the transcription start point of oomycetes [38, 39]. In our investigation, however, presence of this motif did not have a distinct correlation with expression at a given time point in *P*. *sojae*; gene model 260992, for instance, contains this motif but was not expressed *in planta*. More testing would be required before assigning a definitive link between the presence of this motif and PME gene expression.

Intron presence in *Phytophthora* spp., although documented [40, 41], appears to be an infrequent occurrence, with a relative density of 0.62 introns per gene in *P*. *sojae* [26]. Our new study indicates that only six of the 16 PME gene models found (38%) in *P*. *sojae* contain introns of varying sizes; three genes have one intron only, two contain two introns, and the sixth gene model has three introns in its sequence. The percentage of intron-containing, and likely functional PME gene models found in the *P*. *sojae* genome is more or less in agreement with the general estimate of 31.5% suggested by Shen *et al*. [26]. Furthermore, according to our results, the intron density (0.625) closely matches the density previously reported [26]. While these values are smaller than those reported for *Neurospora crassa*, *Cryptococcus neoformans*, *Saccharomyces pombe*, and *Aspergillus nidulans*, they are considerably greater than that found for *S*. *cerevisiae* [42]. Moreover, computational analyses indicate that intron size in *P*. *sojae* is rarely larger than 120bp, with 86bp as the mean size, and most falling between 60 and 90bp [26]. Unfortunately, very little experimental evidence providing insight into the validity and biological characteristics of the introns in *Phytophthora* spp. exists, the few genes studied by Costanzo et al. [40] and Win et al. [41] being the most notable exceptions. The presence of a 236bp intron in gene model 339170 was, therefore, intriguing as it approached the maximum size observed thus far. Our analyses indicate that the 236bp intron in gene model 339170 is actually spliced from the primary transcript and that the annotation is correct (Fig 5). It has been hypothesized that genes in ancestral chromalveolate forms likely had high intron densities (approaching two-thirds of the human intron density), and that the evolutionary history of gene structure in heterokont lineages has been characterized by intron loss [43]. *hytophthora*, however, is estimated to have acquired a considerable number of introns in its evolutionary history while undergoing a less extreme decrease in intron density than its chromalveolate ancestor [43]. Therefore, the experimental confirmation of this intron may give support to the presence, and validity, of similarly sized introns found elsewhere in the *P*. *sojae* genome.

Finally, SignalP analysis provided evidence that all PME gene products in *P*. *sojae* contain a putative signal peptide, which enables them to enter the secretory pathway upon exiting the endoplasmic reticulum (Table 2), and are likely secreted into the apoplast. The ability to be secreted is a critical attribute that would allow PME enzymes to reach the pectin in the host cell wall and play a role during infection.

Understanding how *P*. *sojae* breaks down pectin and enters the soybean host is far from completely understood. This study sought to look into the timing of PME expression in the *P*. *sojae* life cycle. The specific timing of transcription of the PME-coding genes is indicative of when the pathogen’s cell wall degradation process has commenced. As the family of CE-coding genes with the most representation and diversity, PME expression provides a large sample of what the overall expression profile may look like. Knowing the timing of the PME genes expression may have implications in agricultural control mechanisms, as inhibiting pectin breakdown may be a useful strategy aimed at preventing infection by *P*. *sojae*.

## Supporting Information

S1 Appendix DNA sequence of the *Phytophthora sojae* PME genes whose expression was tested by qPCR. (FASTA)

S2 Appendix DNA sequence of all oomycete PME genes used in the phylogenetic analysis. (FASTA)

S1 Table Primer sequences for qPCR and intron analysis in gene model 339170. (DOCX)

S2 Table Relative expression levels of PME-coding genes during infection, as determined by qPCR, in comparison to mycelial expression. (DOCX)
