# Supplementary material for: The Pectin Methylesterase Gene Complement of Phytophthora sojae: Structural and Functional Analyses, and the Evolutionary Relationships with Its Oomycete Homologs
Source: PLoS One. 2015 Nov 6;10(11):e0142096. doi: 10.1371/journal.pone.0142096 (PMC4636286; doi:10.1371/journal.pone.0142096)
Supplement: S1 Table — (DOCX) [file pone.0142096.s003.docx]

**Supporting Information**

**S1 Table. Primer sequences for qPCR and intron analysis in gene model 339170.**

| **Gene ID** | **Primer** | **Primer Sequence (5’-3’)** | **Amplicon Length (bp)** |
| --- | --- | --- | --- |
| 245865 | 245865F 245865R | CTTCGCCGAGGACTTCGT CCAACGTCGTCTTCAAGGAGT | 117 |
| 257384 | 257384F 257384R | TTGAGGAGCAACATGGTGGTG GTGACGATCACCCAAGCCAA | 89 |
| 257416 | 257416F 257416R | GGTGTGGTTGAGGCGGAGAG CTCCTGGCTCCATCGTCGTC | 97 |
| 257622 | 257622F 257622R | GCCGTTGTACCGGTAAGCT GATCCAACGCCCGTGTCACT | 108 |
| 260992 | 260992F 260992R | GCGTTCGACCTTAATCGAAGTC CACGGAGTCGTACGCTGATA | 85 |
| 339170* | 339170F 339170R 339170F12E 339170F1IE | TGACCCACTTTACCGCCGAT CATCGGAGACCGCAACTACC ATGCCACCTCTTGCCAGCCC ATGCCACCGTAAGTCCACT | 114(339170F) 401(339170F12E) 687(339170F1IE) |
| 339194 | 339194F 339194R | GCAGATTTGTTGTAGCAGCCG AGTCGATCGGCAACGGTTC | 109 |
| 340202 | 340202F 340202R | GACCATCCTGCAGGAACTTG CCGAGATTAAGAACGGACGC | 117 |
| 340204 | 340204F 340204R | TGCCATTGCCGATGGAGCT CCGTCGTGTTAACGAGTCCAG | 77 |
| 468280 | 468280F 468280R | CCGTTCAATAGCGGGACTGTC GAAGCTTCCGAACCATTGCG | 118 |
| 491908 | 491908F 491908R | GTCCGACTTGAAGCGGATC TACGCTAGCAACCAGGTGAC | 114 |
| 520304 | 520304F 520304R | GTGCTCCACCGTAGCGATTG TGCCCACCGTGGTAGCTT | 76 |
| 520405 | 520405F 520405R | CCGCTCTGCTGGATCTGAC ATCAAGGACGAGCGCAACTT | 110 |
| 522637 | 522637F 522637R | GACGACTTGTTGAAGTGGCCG GCGATCTCGAGTCGATCGGT | 118 |
| 523081 | 523081F 523081R | GCCCGTTCTTCTGGATATAC TCCCGAGATTACAAGCGGA | 119 |
| 528421 | 528421F 528421R | AGGTAGCTGCCGTCATAGTC GGTCTCGTCACCGGCTTA | 113 |

*All analyses of gene model 339170 used the same reverse primer.
