# Supplementary material for: The Pectin Methylesterase Gene Complement of Phytophthora sojae: Structural and Functional Analyses, and the Evolutionary Relationships with Its Oomycete Homologs
Source: PLoS One. 2015 Nov 6;10(11):e0142096. doi: 10.1371/journal.pone.0142096 (PMC4636286; doi:10.1371/journal.pone.0142096)
Supplement: S2 Table — (DOCX) [file pone.0142096.s004.docx]

**Supporting Information**

**S2 Table. Relative expression levels of PME-coding genes during infection, as determined by qPCR, in comparison to mycelial expression.**

| **Gene ID** | **12hpi** | **24hpi** | **36hpi** | **48hpi** | **72hpi** |
| --- | --- | --- | --- | --- | --- |
| 245865 | 0.001378 | n/d | n/d | 0.000152 | n/d |
| 257384 | 508.1214 | 268.177 | 0.004051 | 68.51818 | n/d |
| 257416 | 0.000133 | 0.401372 | 455.8865 | 0.001262 | 4.31E-05 |
| 257622 | 0.006317 | 6.805192 | 0.060041 | 0.000537 | 0.001227 |
| 260992 | 19.49127 | n/d | n/d | 6.094724 | n/d |
| 339170 | 137.5619 | 1.606559 | n/d | 8.127315 | n/d |
| 339194 | 94.7109 | 0.728968 | 0.35587 | 0.175964 | 2.48E-06 |
| 340202 | 0.010122 | 1.380867 | 0.45263 | 4.03E-05 | 7.25E-06 |
| 340204 | 505.3335 | n/d | 0.001183 | 42.30021 | 0.014077 |
| 468280 | 60.30544 | n/d | 0.236855 | 3.481481 | n/d |
| 491908 | n/d | 0.583711 | 0.048337 | 0.000258 | n/d |
| 520304 | 4.607246 | 5563.084 | 0.000149 | 0.104682 | n/d |
| 520405 | 0.958865 | 0.151501 | 18.52946 | 0.075192 | 2.91E-06 |
| 522637 | 66.13867 | n/d | n/d | 2.632129 | 0.004942 |
| 523081 | 2.71E-05 | 0.744823 | 5.697214 | 2.45E-05 | 1.47E-05 |
| 528421 | 7.743685 | 78.63648 | 0.019123 | 13.61283 | 0.000785 |

n/d: no expression detected
